# 66 Facilitating Health in Schools: The Role of Implementers in the Generation Healthy Kids Program

**DOI:** 10.1093/eurpub/ckae114.066

**Published:** 2024-09-26

**Authors:** Thomas Skovgaard, Jonas Vestergaard Nielsen

**Affiliations:** SDU, Denmark; SDU, Denmark

## Abstract

**Purpose:**

This study investigates the role of local implementation teams in Generation Healthy Kids (GHK), an ongoing multi-component, school-based intervention promoting healthy weight, health, and wellbeing among Danish children aged 6–11.

**Methods:**

The study is one segment of a larger cluster-randomized trial assessing the GHK intervention’s effectiveness and implementation. Semi-structured interviews have been conducted with members of implementation teams, or implementation leads, at the twelve schools randomly assigned to intervention. Interviews were designed to delve into key implementation responsibilities and to identify key challenges and facilitators when promoting the four fundamental intervention components on: diet, physical activity, screen media use, and sleep habits.

**Results:**

With data collection concluding in May 2024, preliminary analyses suggest that successful implementation hinges on securing adequate support and the right competencies among staff members interacting directly with the primary target group, tailoring the program to fit diverse school contexts, and ensuring effective internal and external coordination. Strategic promotion of the intervention among stakeholders is also anticipated to be a critical factor.

**Conclusions:**

The anticipated findings will shed light on organizational and structural elements influencing the effectiveness of multi-layered, school-based health interventions. Implementers – meaning professional personnel at the forefront of execution - emerge as key figures for how well an innovation like GHK is developed and realized. The efficiency of this group is, however, contingent on adequate work infrastructures - including well-organized task arrangements, adequate staffing and support, proactive leadership by school principals, and clear role definitions. The insights gained from this study will inform the design and support of health promotion strategies in schools, with potential implications for policy and practice in child health and wellbeing.

**Funding Source:**

The study is supported via funding from an overall GHK-grant provided by the Novo Nordisk Foundation.

